# The p53-Associated Regulation of ATF4 mRNA Expression Involves p21 and p130 in a Stress-Dependent Manner

**DOI:** 10.3390/cells15181713

**Published:** 2026-09-20

**Authors:** Alexandra Dalina, Maria Shilyaeva, Irina Kovaleva, Peter Chumakov

**Affiliations:** 1 Center for Precision Genome Editing and Genetic Technologies for Biomedicine, Engelhardt Institute of Molecular Biology, Russian Academy of Sciences, 119991 Moscow, Russia; 2 Belozersky Institute of Physico-Chemical Biology, Lomonosov Moscow State University, 119992 Moscow, Russia

**Keywords:** cancer, p53, p21, p130, ATF4, integrated stress response, electron transport chain inhibition, nutrient deprivation, hypoxia

## Abstract

**Highlights:**

**What are the main findings?**

- Chemotherapeutic agents targeting p53 downregulate ATF4 mRNA in a stress-dependent manner.
- p21 and p130 contribute to the specific p53-dependent downregulation of ATF4 mRNA upon mitochondrial dysfunction.
- ATF4 mRNA levels are reduced under hypoxic conditions and partially restored by p130 knockdown.

**What is the implication of the main findings?**

- This study suggests the existence of a new link between p53 and ATF4.

**Abstract:**

p53-dependent signaling and the integrated stress response (ISR) are major stress response programs that coordinate cellular metabolism and cell fate decisions. While p53 activation often promotes cell cycle arrest and cell death, the ISR can support either adaptive survival or cell death depending on the cellular context. Although increasing evidence indicates the crosstalk between these pathways, the underlying mechanisms remain incompletely understood. Here, we show that p53 activation is associated with reduced ATF4 mRNA expression under basal conditions and during selected metabolic stresses, including mitochondrial dysfunction. Knockdown experiments have demonstrated that p21 and p130 contribute to this response in a stress-dependent manner. Our findings identify a context-dependent link between p53 signaling and ATF4 mRNA regulation and suggest that p53, p21, and p130 may influence the ATF4-dependent branch of the ISR during cellular stress.

## 1. Introduction

p53 is a tumor suppressor involved in multiple processes that determine cell fate, such as cell cycle arrest and death, and regulate cellular homeostasis. It is induced in response to a wide variety of stresses and transcriptionally activates a number of target genes in response to a wide spectrum of stress stimuli, including DNA damage, oxidative stress and various metabolic stresses such as nutrient deprivation, hypoxia, and mitochondrial inhibition [1,2,3,4,5]. Its level and activity are tightly regulated primarily at the protein level by its interactions with other proteins, post-translational modifications (PTMs), and the modulation of its degradation.

p53 upregulation and activation have been shown to occur in the presence of the inhibitor of the mitochondrial electron transport chain complex III myxothiazol, in contrast to other inhibitors of ETC complexes, such as complex I inhibitor piericidin A, complex II inhibitor TTFA, and complex IV inhibitor KCN [6]. p53 begins to accumulate 6 h after treatment with myxothiazol, with its level peaking 12 h after the treatment [6]. Transcriptomic analysis showed that a short-term (4–5 h) treatment with myxothiazol elevated the mRNA levels of the key player of the integrated stress response (ISR) ATF4, which gradually declined along with p53 accumulation [7].

The upregulation of the transcription factor ATF4 is one of the hallmarks of the integrated stress response (ISR), an evolutionarily conserved homeostatic program that orchestrates a number of metabolic processes and may lead to different outcomes depending on the context. The major ISR event is the phosphorylation of the α subunit of the translation initiation factor 2 (eIF2α) in response to metabolic stresses, resulting in global translation inhibition. This promotes the translation of ATF4 and two other ISR-related genes, CHOP and GADD34 [8]. In the presence of various metabolic stresses, such as amino acid and glucose deprivation, endoplasmic reticulum (ER) stress, mitochondrial dysfunction, and oxidative stress, ATF4 transcriptionally activates or represses numerous genes implicated in pro-survival as well as suicidal programs: amino acid transporters; genes involved in autophagy, translation, and mitochondrial function regulation; and proapoptotic genes [8,9]. The ATF4-dependent gene expression program depends on its post-translational modifications, heterodimerization partners, and histone modifications near the target genes [8]. Most studies are primarily focused on the regulation of ATF4 at the protein level via its translation and degradation; however, data on the regulation of ATF4 transcription are scarce, so we aimed at filling in this gap.

Since the mechanism behind p53 induction relies on the inhibition of the enzyme dihydroorotate dehydrogenase (DHODH), involved in the pyrimidine biosynthesis pathway [6], co-treatment with uridine upregulates ATF4, similarly to short exposures to myxothiazol or complex I inhibition by piericidin A [8]. p53 has been shown to be capable of the indirect transcriptional repression of genes via the p21-DREAM-CDE/CHR pathway [10,11]. In brief, p53 transcriptionally activates the cyclin-dependent kinase inhibitor p21^Cip1/Waf1^ (CDKN1A), which, in turn, facilitates the hypophosphorylation of the pRb-related pocket proteins p130 and p107 [10,12], promoting the assembly of the inhibitory complex DREAM (named after its components: DP, RB-like, E2F4, and MuvB proteins). The binding of DREAM to the cell cycle-dependent element (CDE) and cell cycle genes homology region (CHR) in the promoters of target genes, such as genes involved in the G2/M transition, results in their transcriptional repression. We suggested that this mechanism at least partially might be involved in the ATF4 transcriptional suppression by p53 that takes place during DHODH inhibition. To address this, we examined whether the observed effect was limited to this particular stress or represented a common mechanism of ATF4 regulation, and next, we assessed ATF4 mRNA levels in HCT116 colon cancer cells with wild-type p53 during exposure to a number of p53-inducing agents in the absence or presence of the stimuli that cause ATF4 activation, including ETC inhibitors, nutrient deprivation, and compounds that trigger ER stress. We studied the role of the p53-p21-DREAM pathway in ATF4 mRNA regulation by estimating ATF4 mRNA levels in HCT116 cells with p53, p21, or p130 downregulation and revealed the contribution of each protein in maintaining ATF4 transcription under various stress conditions. Taken together, our findings shed light on the context-dependent roles of p53, p21, and p130 in the regulation of ATF4 expression and thus bring to the surface new aspects of the interplay between p53-dependent signaling and ISR.

## 2. Materials and Methods

### 2.1. Cell Lines and Treatments

HCT116 human colon cancer and HEK293T human embryonic kidney cells (ATCC, verified by STR, tested for the absence of mycoplasma using the Myco Real-time qPCR kit (Evrogen, Moscow, Russia, cat# MR004)) were cultured in DMEM (high glucose, ServiceBio, Wuhan, China, cat# G4514), containing 10% fetal bovine serum (HyClone, Cytiva, Marlborough, MA, USA, cat# SH30088.03), 4 mM L-alanyl-L-glutamine (Gibco, Thermo Fisher Scientific, Waltham, MA, USA; cat# 35050061) and penicillin–streptomycin (100 units/mL, PanEco, Moscow, Russia). Cells were maintained at 37 °C with 5% CO2 in a humidified atmosphere.

### 2.2. Preparation of Stable Knockdown Cell Lines

To generate cells with the stable knockdown of p53, p21, or p130, the following shRNAs were used: GACTCCAGTGGTAATCTAC, CTCAGTTTGTGTGTCTTAATT, or GTGTTCTAATCGTAAAGAAC, respectively. Lentiviral constructs were created by introducing oligonucleotides, each containing an shRNA sequence, a complementary sequence, a loop sequence, and BamHI and EcoRI sticky ends, into a pLSL lentiviral vector containing puromycin or hygromycin resistance genes as selection markers. Stable HCT116 cell lines with p53, p21, or p130 knockdown were obtained using lentiviral transduction; HEK293T cells were co-transfected with the vector pLSLP-shRNA and packaging plasmids (pCMV-Gag, pCMV-Rev, pCMV-VSV-G) using the GenJect 40 transfection reagent (Molecta, Moscow, Russia); and lentiviral particles were harvested 36 h after transfection. Transduced HCT116 cells were selected with 1 µg/mL puromycin or 250 µg/mL hygromycin. RNA interference efficiency was determined with RT-qPCR and/or Western blot analysis.

### 2.3. RT-qPCR

Total RNA was isolated using the ExtractRNA reagent (Evrogen; cat# BC032) according to the manufacturer’s protocol followed by precipitation with 3 M sodium acetate (pH 4.8). RNA concentration and purity were determined using a NanoDrop 2000 spectrophotometer (Thermo Fisher Scientific), and RNA integrity was confirmed by agarose gel electrophoresis. 500 ng of total RNA was reverse-transcribed with random hexamers (with a final concentration of 0.5 μM) using the Mint RT kit (Evrogen; cat# SK001) according to the manufacturer’s instructions, and obtained cDNA was further diluted in mQ water. Reverse transcription conditions were standardized across experiments by reference to a mixed control sample comprising cDNA from multiple controls. RT-qPCR was performed with the HS-Taq SYBR Green master mix (Evrogen, cat# PK147) using a Bio-Rad CFX96 C1000 Touch Real-Time PCR detection system (Bio-Rad Laboratories, Hercules, CA, USA) under the following cycling conditions: initial heat denaturation at 95 °C for 3 min, followed by 40 cycles of denaturation at 95 °C for 10 s, annealing at 64 °C for 15 s, and elongation at 72 °C for 15 s coupled with fluorescence measurements. A melt curve analysis was performed after the final 3 min elongation at 72 °C. The primers used are listed in Table 1:

At least four biological replicates were analyzed, each in technical duplicate. The relative expression of target genes was normalized by the geometric mean of the TBP and TUBGCP2 reference genes using the Pfaffl method. Statistical significance was calculated using an ANOVA and Tukey’s post hoc test. Data are shown as the mean ± SEM. The following marks were used for *p*-values: *p* < 0.05 (*), *p* < 0.01 (**), and *p* < 0.001 (***).

### 2.4. Western Blot

For Western blot analysis, cells were washed twice with PBS and lysed in M-PER protein extraction reagent (Thermo Fisher Scientific, cat# 78501) according to the manufacturer’s instructions. Samples with equal amounts of total protein were heated at 95 °C for 5 min in the Laemmli sample buffer and separated on a 12% SDS–polyacrylamide gel (20 min at 80 V, then approximately 1 h at 100 V). Proteins were transferred to a 0.2 μm Immobilon-P PVDF membrane (Merck Millipore, Billerica, MA, USA; cat# IPVH00010) at 40 mA for 12 h. Membranes were blocked with 4% non-fat milk in PBST (PBS with 0.05% Tween 20, Sigma-Aldrich, St. Louis, MO, USA) for 1 h at room temperature and incubated with the following primary antibodies overnight at 4 °C: p53 (DO-1, Santa Cruz Biotechnology, Dallas, TX, USA; cat# sc-126) at a dilution of 1:1000, p21 (12D1, Cell Signaling Technology, Cell Signaling Technology, Danvers, MA, USA; cat #2947) at a dilution of 1:2500, and vinculin (EPR8185, Abcam, Cambridge, UK; cat# ab129002) at a dilution of 1:5000. Then, membranes were washed with PBST and incubated with either anti-rabbit (Bio-Rad Laboratorie; cat# 1706515) or anti-mouse (Bio-Rad Laboratories; cat# 1706516) HRP-conjugated secondary antibodies for 1 h at room temperature. Detection was performed with either Immobilon ECL (Merck Millipore; cat# WBULS0100) or ECL Clarity Max (Bio-Rad Laboratories; cat# 1705062) detection reagents.

## 3. Results

### 3.1. ATF4 mRNA Expression Is Suppressed by Various p53-Inducing Agents

The inhibition of ETC complex III by myxothiazol has been previously found to suppress ATF4 mRNA expression in HCT116 colon cancer cells with wild-type p53 but not in p53-deficient cells [13]. Therefore, we hypothesized that p53 induction by other stress stimuli might also repress ATF4 and estimated ATF4 mRNA levels in HCT116 cells exposed to a number of low-molecular compounds that induce p53 via different mechanisms: doxorubicin (topoisomerase II inhibitor), cisplatin (induces DNA breaks), topotecan and camptothecin (topoisomerase I inhibitors), nutlin-3a and RG7388 (specific p53 inducers [14]), HZ00 and PALA (inhibitors of the de novo pyrimidine biosynthesis pathway enzymes, DHODH and CAD, respectively [15,16]), and 5-fluorouracil (thymidylate synthase inhibitor). We evaluated p53 activity by assessing the mRNA levels of the specific p53 target TP53INP1 as well as p21, a p53 target that might be involved in p53-dependent ATF4 repression.

**Figure 1:**
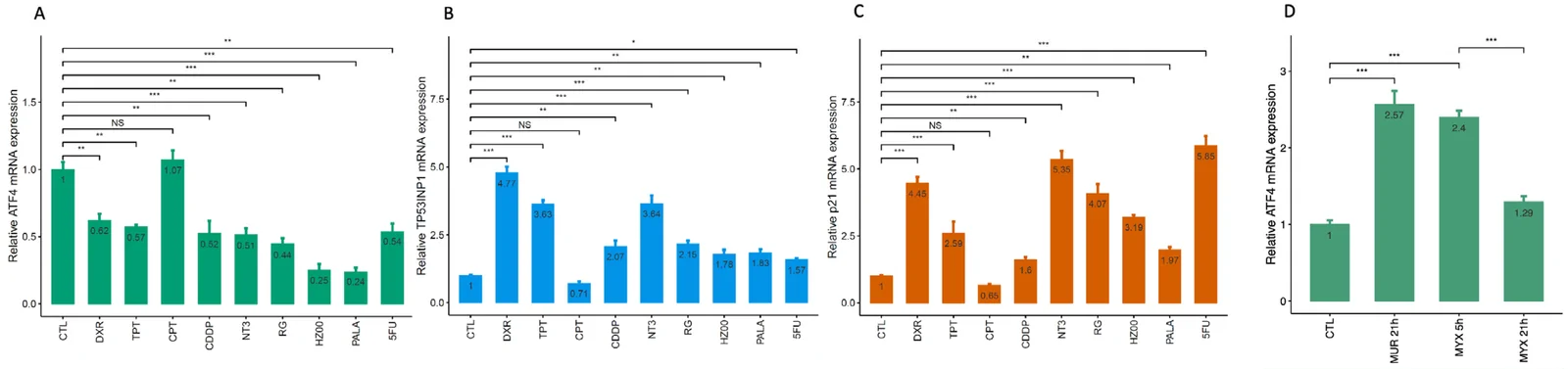
(**A**–**C**) Various p53-inducing agents suppress ATF4 transcription. mRNA levels of ATF4 (**A**), TP53INP1 (**B**), and p21 (**C**) in HCT116 cells with wild-type p53 (GFPsh) after 21 h incubation with 0.5 µM doxorubicin (DXR), 0.5 µM topotecan (TPT), 400 nM camptothecin (CPT), 3 µg/mL cisplatin (CDDP), 5 µM nutlin-3a (NT3), 200 nM RG7388 (RG), 10 nM HZ00, 400 µM PALA, 10 µg/mL 5-fluorouracil (5FU), or mock control (CTL). (**D**) Influence of ETC inhibition by myxothiazol (2 µM) on ATF4 mRNA levels at various time points alone (MYX) or in combination with uridine (1 mM), which prevents p53 activation (MUR). All mRNA levels were normalized by control. Results are presented as mean ± SEM from three experiments with 4 biological replicates, control samples and doxorubicin, which were averaged across all experiments. One-way ANOVA, followed by Tukey’s post hoc test, was used. * *p* < 0.05, ** *p* < 0.01, *** *p* < 0.001, NS—non-significant.

All tested compounds reduced the basal ATF4 mRNA level (in the absence of stresses that activated ATF4 transcription), except camptothecin (Figure 1A); although its mechanism of action is the same as that of topotecan, it did not elevate the mRNA levels of the p53 targets TP53INP1 and p21 (Figure 1B,C). We would like to highlight that ATF4 was also transcriptionally repressed by nutlin-3a and RG7388, compounds that specifically induce p53 by hindering its interaction with the ubiquitin ligase MDM2 and thus preventing its proteasomal degradation [16], which is in line with the published transcriptomic data [17].

We also tested paclitaxel, which causes mitotic arrest via microtubule stabilization, and, in contrast, it elevated the ATF4 mRNA level, which accords with the previous data [18]. This effect is likely to involve ATF4 transactivation via a different mechanism, as is the case with myxothiazol [6,13]. ATF4 mRNA levels were significantly elevated in the absence of p53 induction (after a 5 h incubation or upon supplementation with uridine), while longer incubations, accompanied by p53 accumulation and activation, caused a decline in ATF4 mRNA levels (Figure 1D, also shown in [13]). Taken together, our results imply that p53 activation mediated by different mechanisms can suppress basal ATF4 mRNA expression.

### 3.2. Suppression of ATF4 mRNA Expression by p53 Depends on Mechanism of ATF4 Activation

**Figure 2:**
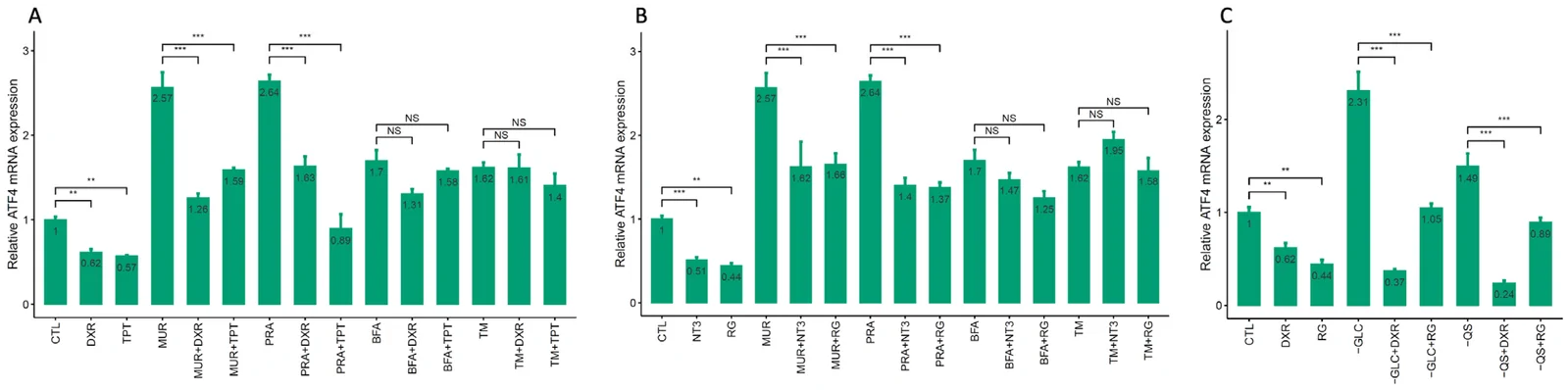
(**A**,**B**) p53 activation by doxorubicin and topotecan (**A**) as well as specific activation by nutlin-3a and RG7388 (**B**) suppresses ATF4 mRNA levels in absence of ATF4 activators as well as in presence of ETC inhibitors piericidin A and myxothiazol in combination with uridine, in contrast to inducers of ER stress brefeldin A and tunicamycin. (**C**) Doxorubicin and RG7388 suppress ATF4 mRNA levels during glucose starvation and deprivation of glutamine and FBS. (**A**,**B**) mRNA levels of ATF4 in HCT116 cells with wild-type p53 (GFPsh) treated with 0.5 µM doxorubicin (DXR), 0.5 µM topotecan (TPT) (**A**); 5 µM nutlin-3a (NT3), 250 nM RG7388 (RG) (**B**) alone or in combination with 250 nM piericidin A (PRA), 2 µM myxothiazol + 1 mM uridine (MUR), 5 µg/mL tunicamycin (TM), 25 µg/mL brefeldin A (BFA) (**A**,**B**). (**C**) mRNA levels of ATF4 in HCT116 cells with wild-type p53 (GFPsh) treated with 0.5 µM doxorubicin (DXR) or 250 nM RG7388 (RG) in the control medium, in the medium without glucose supplemented with 10% FBS, 4 mM glutamine, and 10 mM galactose (-GLC), or medium without glutamine and FBS supplemented with 25 mM glucose (-QS). Cells were incubated with tunicamycin and brefeldin A for 5 h and with all other compounds for 21 h. Relative expression was normalized by mock control (CTL). Results are presented as mean ± SEM from three experiments with 4 biological replicates. Two-way ANOVA, followed by Tukey’s post hoc test, was used. ** *p* < 0.01, *** *p* < 0.001, NS—non-significant.

Next, we determined whether the p53-dependent suppression of ATF4 mRNA also took place in the presence of stresses activating ATF4. To test this hypothesis, we assessed the effects of doxorubicin and topotecan in the presence of piericidin A and a combination of myxothiazol with uridine, which inhibit ETC complexes I and III without p53 induction and lead to ATF4 transactivation (Figure 2A), as well as tunicamycin and brefeldin A, compounds that trigger ER stress. The accumulation of p53 and its targets TP53INP1 and p21 was confirmed by qPCR and Western blotting (Supplementary Figures S1 and S2). Both doxorubicin and topotecan reduced ATF4 mRNA levels either in the absence of stresses that activated ATF4 or in the presence of the ETC inhibitors. However, they failed to downregulate ATF4 upon exposure to the ER stress-inducing agents brefeldin A and tunicamycin, although they did not affect p53 activation, as reflected by TP53INP1 and p21 mRNA levels (Supplementary Figure S1). The same effects were observed for the specific p53 inducers, nutlin-3a and RG7388 (Figure 2B). This implies that the p53-dependent suppression of ATF4 could be disrupted by certain stimuli that trigger ATF4 activation, such as ER stress.

We also checked whether the p53-inducing agents under scrutiny could downregulate ATF4 mRNA during glucose or glutamine starvation, which have been shown to activate ATF4 at both the mRNA and protein levels [19,20]. Glucose starvation and the simultaneous deprivation of glutamine and fetal bovine serum caused a pronounced increase in ATF4 mRNA, while RG7388 completely prevented ATF4 upregulation, and doxorubicin even further reduced ATF4 mRNA levels (Figure 2C). Together with the previous data, the latter observation serves as evidence that p53 is involved in the regulation of ATF4 mRNA expression during mitochondrial dysfunction and glutamine or glucose starvation. As for doxorubicin, it is likely to utilize both p53-dependent and independent mechanisms to suppress ATF4 mRNA expression under nutrient deprivation conditions.

### 3.3. The Contribution of p21 and p130 to ATF4 mRNA Downregulation by p53-Inducing Agents

**Figure 3:**
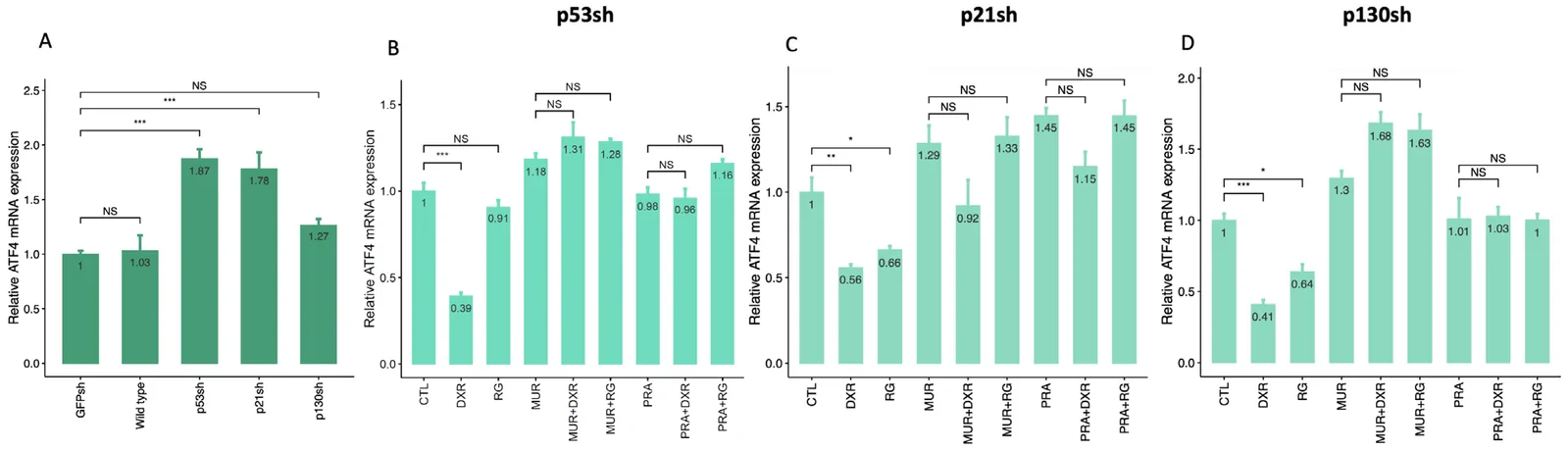
(**A**) Knockdown of p53 or p21 elevates ATF4 mRNA levels in absence of ATF4 activators, in contrast to p130. (**B**–**D**) p53 knockdown completely prevents ATF4 downregulation by RG7388 but not by doxorubicin in absence of ATF4 activators, in contrast to p21 and p130 knockdown, while knockdown of either p53, p21, or p130 prevented RG7388- and doxorubicin-induced ATF4 mRNA downregulation in presence of ETC inhibitors. mRNA levels of ATF4 were estimated in HCT116 cells expressing GFPsh (**A**), p53sh (**B**), p21sh (**C**), or p130sh (**D**), treated with 0.5 µM doxorubicin (DXR) or 250 nM RG7388 (RG) alone or in combination with 250 nM piericidin A (PRA) or 2 µM myxothiazol + 1 mM uridine (MUR) for 21 h. Relative expression was normalized by mock control (CTL). Results are presented as mean ± SEM from three experiments with 4 biological replicates. Two-way ANOVA, followed by Tukey’s post hoc test, was used. * *p* < 0.05, ** *p* < 0.01, *** *p* < 0.001, NS—non-significant.

The role of p53 induction in the observed ATF4 downregulation was further assessed using p53 knockdown. RNAi efficiency was estimated by qPCR (Supplementary Figure S3B) and Western blotting (Supplementary Figure S4A). ATF4 mRNA levels in cells with p53 knockdown were higher compared to those in control GFPsh cells (Figure 3A and Supplementary Figure S3A). p53 knockdown prevented ATF4 downregulation by RG7388, which serves as convincing evidence supporting the contribution of p53 to ATF4 suppression (Figure 3B and Supplementary Figure S3A). Meanwhile, although doxorubicin, similarly, did not downregulate ATF4 mRNA during ETC inhibition, it still decreased basal ATF4 mRNA levels in p53-deficient cells, which can be explained by the fact that it can upregulate certain p53 targets, such as p21, in a p53-independent manner [21]. Taken together, these results suggest that p53 maintains basal ATF4 mRNA levels and fine-tunes them upon mitochondrial dysfunction.

Since the mechanism of the indirect p53-dependent transrepression involves p21 and p130 [10], we compared basic ATF4 mRNA levels under control conditions (in the absence of stresses) in GFPsh, p53sh, p21sh, and p130sh cell lines (Figure 3A). RNAi efficiencies for p21 and p130 were assessed by RT-qPCR and/or Western blotting (Supplementary Figures S3F and S4B,D). Like p53, p21 knockdown significantly elevated ATF4 mRNA levels (and even to a greater extent), which was not the case for p130 knockdown. This implies that, similarly to p53, p21 is involved in maintaining basic ATF4 levels.

We next examined ATF4 mRNA expression in HCT116 cells with p21 or p130 knockdown following treatment with doxorubicin or RG7388 (Figure 3C,D and Supplementary Figure S3D,G). In p21sh or p130sh cells exposed to ETC inhibitors, neither doxorubicin nor RG7388 reduced ATF4 mRNA expression. In contrast, under basal conditions, the depletion of p21 or p130 did not prevent the reduction in ATF4 mRNA induced by either doxorubicin or RG7388. These results indicate that p21 and p130 are not individually required for the reduction in ATF4 mRNA associated with p53 activation in the absence of ATF4-inducing stress. However, under mitochondrial respiration inhibition, the loss of either p21 or p130 abolished the reduction in ATF4 mRNA associated with RG7388 and doxorubicin treatment. Thus, p21 and p130 contribute to the p53-associated regulation of ATF4 mRNA expression during mitochondrial stress.

### 3.4. Regulation of ATF4 mRNA Expression Under Hypoxic Conditions

**Figure 4:**
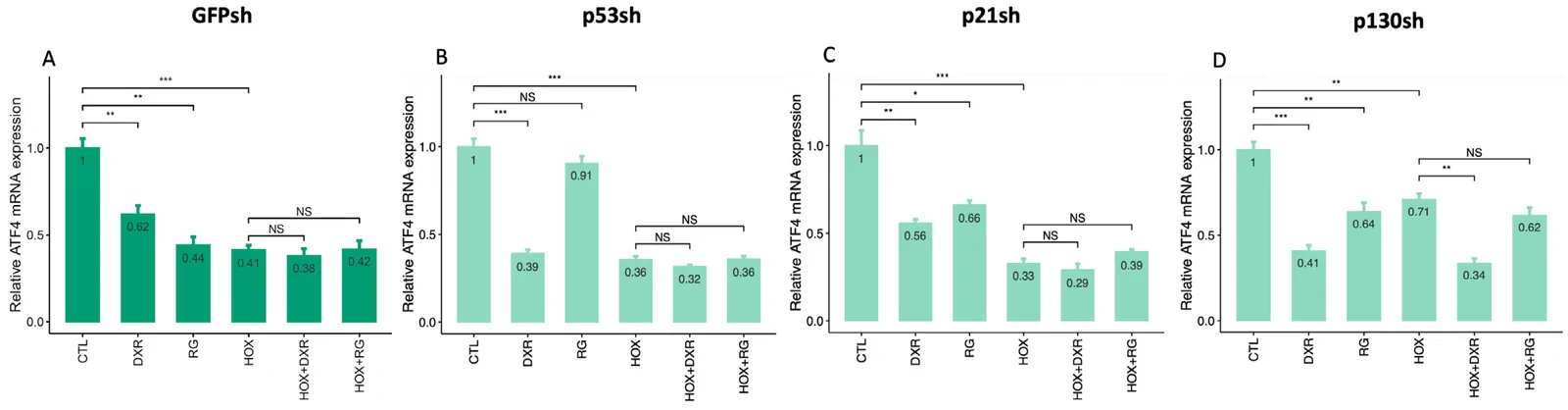
p53 activation by doxorubicin and RG7388 has no effect on ATF4 mRNA levels under hypoxic conditions, which alone downregulates ATF4 mRNA; knockdown of p53 or p21 does not affect ATF4 mRNA levels during hypoxia, whereas p130 knockdown alleviates p53-dependent ATF4 mRNA suppression. mRNA levels of ATF4 were estimated in HCT116 cells expressing GFPsh, p53sh, p21sh, and p130sh (**A**); p53sh (**B**); p21sh (**C**); or p130sh (**D**), treated with 0.5 µM doxorubicin (DXR) or 250 nM RG7388 (RG) during normoxia or hypoxia incubated for 21 h. Relative expression was normalized by normoxic mock control (CTL). Results are presented as mean ± SEM from three experiments with 4 biological replicates. Two-way ANOVA, followed by Tukey’s post hoc test, was used. * *p* < 0.05, ** *p* < 0.01, *** *p* < 0.001, NS—non-significant.

Hypoxia is another stress stimulus which is known to affect ATF4 expression [19,21,22]. There is solid evidence of hypoxia-induced ATF4 upregulation at the protein level; however, it remains unclear whether it transcriptionally activates ATF4. To determine the impact of hypoxia on ATF4 mRNA expression, we assessed ATF4 mRNA levels in cells treated with either doxorubicin or RG7388 under hypoxic conditions (Figure 4A). Unexpectedly, hypoxia further downregulated ATF4 mRNA in control cells (Figure 4A) and p53 transcriptional activity as indicated by the expression of its specific target TP53INP1 (Supplementary Figure S5B, GFPsh), while it did not affect p21 mRNA levels (Supplementary Figure S5C, GFPsh). p53 and p21 knockdown had no further effect on the ATF4 mRNA content during hypoxia (Figure 4B,C and Supplementary Figure S5A,D), while p130 knockdown, unlike p53 and p21, elevated the ATF4 mRNA levels under hypoxic conditions alone or in the presence of RG7388, its effect being much higher compared to normoxia (Figure 4D and Supplementary Figure S5G). Of note, treatment with doxorubicin prevented ATF4 mRNA upregulation by p130 knockdown, indicating that its mechanism of action did not depend on either of these proteins. Taken together with the previously obtained results, this implies the possible role of p130—in contrast to p21 and p53—as an important regulator of ATF4 during hypoxia.

## 4. Discussion

Accumulating evidence indicates extensive crosstalk between p53- and ATF4-dependent signaling networks, two major stress-responsive hubs that coordinate cellular metabolism and cell fate decisions. Both factors can induce pro-survival or proapoptotic gene expression programs depending on the type, intensity, and duration of stress. p53 and ATF4 are regulated predominantly at the protein level through post-translational modifications, proteolysis, and translational control, thereby enabling rapid stress responses [8,9,23,24,25]. The selection of p53- and ATF4-responsive genes is further shaped by post-translational modifications, interacting partners, and chromatin context [8,26]. Both proteins respond to overlapping stressors, including oxidative stress, hypoxia, and nutrient deprivation [9,25], and numerous additional proteins intersect with both signaling networks.

**Figure 5:**
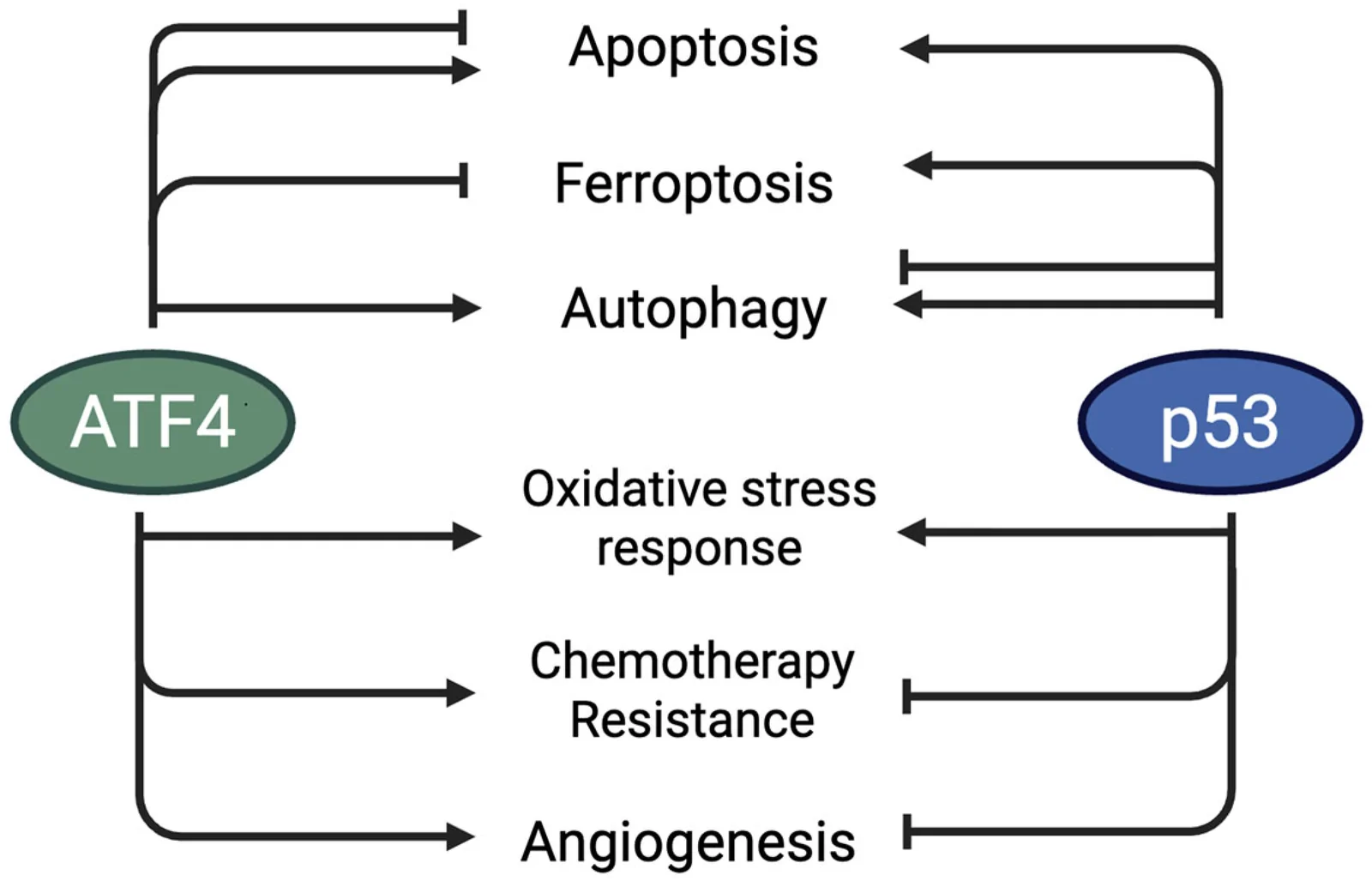
Schematic model of functional crosstalk between ATF4 and p53.

p53 and ATF4 also share several reported transcriptional targets, including SESN2, CDKN1A (p21), PUMA, NOXA, DDIT4 (REDD1), DR5, and ATF3 [5,7,9,23,27,28,29,30,31,32,33]. Accordingly, ATF4 can induce selected proapoptotic genes in cells lacking functional p53 under particular conditions p53 [30,34,35]. Both pathways also converge on mTOR signaling through targets such as SESN2 and REDD1 [27,35]. Although ATF4-dependent programs often support metabolic adaptation and survival, sustained ATF4 activity can also promote cell death; similarly, p53 can induce growth arrest, apoptosis, or adaptive metabolic responses. Their crosstalk may therefore help balance stress adaptation with cell death-associated responses (Figure 5). For example, ATF4 has been reported to promote VEGF expression [8] and stimulate glycolysis via its target PHGDH and pro-survival autophagy, whereas p53 can limit VEGF expression in an Rb-dependent context and suppress glycolysis through TIGAR, while also supporting autophagy, glutamine metabolism, and mitochondrial respiration [8,36,37,38]. These observations underscore the importance of defining the mechanisms that connect p53 activity with ATF4 mRNA regulation (Figure 5).

ATF4 has been reported to affect p53 abundance and activity through the regulation of its negative regulator MDM2 [39] and miR-145 [40]. However, data on the possible regulation of ATF4 by p53, rather than the coregulation of cell metabolism, are scarce, so we studied a panel of chemotherapeutic drugs that induce p53 via different mechanisms as well as two specific p53-inducing agents, nutlin-3a and RG7388, and found out that all tested compounds, except camptothecin, which failed to activate p53, indeed, suppressed ATF4 mRNA expression. The fact that ATF4 downregulation by RG7388 was prevented by p53 knockdown confirmed the role of p53 in maintaining ATF4 mRNA levels. However, doxorubicin downregulated ATF4 mRNA even in p53-deficient cells, indicating p53-independent mechanisms being involved.

p53 primarily regulates gene expression by binding p53 response elements (p53REs) within the regulatory regions of target genes, thereby promoting transcriptional activation. The mechanisms of p53-dependent gene repression are less well characterized but can include non-coding RNAs and the p21–DREAM–CDE/CHR pathway (reviewed in [41]). The currently known p53-regulated miRNAs and miRNAs reported to target ATF4 do not substantially overlap; therefore, it remains to be determined whether a miRNA-mediated pathway links p53 activity to ATF4 mRNA expression [42,43]. Among lncRNAs, p53 negatively regulates MALAT1, whose expression correlates with ATF4 mRNA levels in colon cancer cells [43,44,45,46]. Whether MALAT1 contributes to the p53-associated decrease in ATF4 mRNA observed here remains unknown.

One study suggested that p53 may regulate target genes differentially according to p53RE sequence [47]. More recently, p53 was reported to repress the *SLC7A11* and *MTHFD2* [48,49] genes. However, both genes are also direct ATF4 targets [8] and could therefore be indirectly downregulated following reduced ATF4 expression. The diversity of p53 isoforms, the ability of other p53 family proteins to recognize related DNA sequences [50,51,52,53,54,55,56,57], and the activity of p53-interacting regulators, including the ubiquitin ligases MDM2 and Pirh2, further increase the complexity of p53-dependent gene regulation [58,59].

We used the JASPAR database [60] to identify putative binding motifs for p53, E2F4, MYC, MuvB, and the DREAM component LIN54 within the analyzed ATF4 promoter region. We identified several predicted MuvB-, LIN54-, and E2F4-related motifs but no canonical p53RE. We also did not identify canonical motifs for p63 or p73, which recognize DNA sequences related to p53REs. These in silico results do not establish factor binding, exclude distal regulatory elements, or prove the functional relevance of the predicted motifs. Nevertheless, they make direct regulation through a canonical p53RE in the analyzed promoter region less likely and support the consideration of indirect mechanisms.

In addition to E2F4- and NRF2-related motifs, the analyzed ATF4 promoter region contained multiple predicted CREB1 and C/EBPβ binding sites. C/EBPβ-LIP has been reported to repress ATF4 expression after UV exposure, a condition that also activates p53 [61,62]. LIP is an inhibitory C/EBPβ isoform that competes with the transcriptionally active LAP isoforms; thus, the LIP/LAP ratio can influence C/EBPβ-dependent gene regulation. p53 overexpression was reported to reduce C/EBPβ expression, particularly that of LAP isoforms, through mechanisms independent of its transcriptional activity [63]. Although LIP was also reduced in that study, the relative effect on individual C/EBPβ isoforms may be cell type- and stress-dependent. Therefore, a p53–C/EBPβ interaction could potentially contribute to ATF4 mRNA suppression, including the p53-independent effect of doxorubicin, but this hypothesis requires direct experimental testing.

It should be emphasized that we measured total steady-state ATF4 mRNA. Therefore, the observed reduction in ATF4 mRNA after RG7388 or doxorubicin treatment may reflect decreased transcription, reduced mRNA stability, enhanced RNA degradation, or a combination of these processes. In [61], ATF4 mRNA stability was assessed using an actinomycin D chase. The authors concluded that changes in ATF4 mRNA abundance after UV exposure or treatment with the ER stress inducer thapsigargin were not explained by the altered mRNA half-life and were instead consistent with altered transcription. However, using actinomycin D to evaluate p53-dependent responses is difficult to interpret because actinomycin D itself activates p53. Alternative approaches, including metabolic RNA labeling followed by the click chemistry-based detection of nascent or newly synthesized RNA, would be more suitable for resolving the contribution of transcription and mRNA stability to the p53-associated regulation of ATF4 expression.

RNA modifications can influence mRNA stability, translation efficiency, and alternative translation initiation. ATF4 mRNA stability has been reported to depend on pseudouridylation and methylation [64,65,66,67,68]. Such mechanisms may contribute to the loss of p53-associated ATF4 mRNA suppression during ER stress. Although p53 can regulate epitranscriptomic writer, reader, and eraser proteins [69], available evidence is heterogeneous, and it remains unclear whether these pathways are involved in the p53-associated regulation of ATF4 mRNA abundance.

Although transcriptomic data from [17] showed that the knockout of the DREAM component LIN37 did not prevent nutlin-3a-associated ATF4 mRNA downregulation, we examined whether p21 and p130 could nevertheless contribute to this response. p130 can function in repressive p130/E2F4 complexes, including complexes involving HDAC1 and ΔNp73α [70], and can recruit the CtBP corepressor [71]. p21 knockdown markedly increased basal ATF4 mRNA levels, whereas p130 knockdown had no comparable effect. However, neither p21 nor p130 depletion prevented the reduction in ATF4 mRNA induced by RG7388 or doxorubicin under basal conditions. These observations indicate that the p53-associated regulation of ATF4 mRNA in unstressed cells is not fully explained by p21 or p130 and may involve additional pathways.

The mode of ATF4 induction appears to influence the p53-associated suppression of ATF4 mRNA expression. DNA-damaging agents and specific p53 activators reduced ATF4 mRNA expression in cells exposed to ETC inhibitors or nutrient deprivation but did not reduce ATF4 mRNA during ER stress. Thus, ER stress appears to override, or otherwise disrupt, the p53-associated negative regulation observed under the other conditions.

One possible explanation is that ER stress induces positive feedback circuits involving ISR-responsive transcription factors or factors that regulate ATF4 mRNA stability. For example, XBP1 may contribute to ATF4 expression. XBP1 and its upstream regulator IRE1α are activated during ER stress in colon cancer cells, and XBP1 has been implicated in ATF4 transactivation [72,73]. p53 may potentially reduce XBP1 expression through miR-34a, which directly targets XBP1 mRNA and could contribute to the p53-associated decrease in ATF4 mRNA. During strong ER stress, however, XBP1 and IRE1α may form a negative feedback circuit involving the inhibition of miR-34a transcription, which could weaken p53-dependent miR-34a regulation and thereby account for the lack of ATF4 mRNA suppression in this setting [74]. This model remains speculative and requires direct testing. CREB is another potential contributor because it can bind the ATF4 promoter in HEK293 cells [75,76].

The knockdown of p53, p21, or p130 prevented the decrease in ATF4 mRNA induced by RG7388 and doxorubicin in cells exposed to ETC inhibitors. These data suggest that p53, p21, and p130 contribute to the regulation of ATF4 mRNA abundance during mitochondrial dysfunction. One possible consequence is that this regulation limits excessive ATF4 protein accumulation during prolonged intrinsic stress; however, ATF4 protein abundance and ISR activity were not measured in the present study. Future work should therefore determine how the p53-dependent regulation of ATF4 mRNA affects ATF4 protein expression, ISR intensity, and ATF4-dependent chemoresistance under nutrient deprivation and mitochondrial dysfunction.

Finally, we examined hypoxia, another stress condition known to activate ATF4-dependent programs. Although hypoxia can increase ATF4 protein levels through translational control, there are limited data on the regulation of ATF4 mRNA abundance, transcription, or stability under hypoxic conditions. Unexpectedly, hypoxia significantly reduced ATF4 mRNA levels independently of p53 or p21 status. Based on published evidence of the hypoxia-induced translational upregulation of ATF4 in other experimental systems [19,21,22], reduced ATF4 mRNA abundance could potentially contribute to limiting excessive or prolonged ATF4 activation. However, ATF4 protein abundance was not measured in the present model; therefore, this interpretation remains speculative. Further studies are needed to define how hypoxia coordinately regulates ATF4 transcription, mRNA stability, translation, protein turnover, and downstream ISR activity.

p130 knockdown partially alleviated the reduction in ATF4 mRNA induced by hypoxia alone and by RG7388 treatment under hypoxic conditions. In contrast, doxorubicin reduced ATF4 mRNA levels in p130-deficient cells, indicating the involvement of additional p130-independent mechanisms. The effect of p130 knockdown was more pronounced under hypoxia than under normoxia, suggesting that p130 contributes to the regulation of ATF4 mRNA abundance under hypoxic conditions. p130/E2F4 complexes have been reported to repress RAD51, a gene involved in homologous recombination repair, during hypoxia [77]. A similar p130/E2F4-dependent mechanism may contribute to ATF4 mRNA regulation under hypoxia and, consequently, to cellular adaptation to low oxygen. However, this possibility remains to be tested directly. In addition, p107 can compensate for some p130 functions upon p130 depletion [12]. Therefore, further studies using complementary loss-of-function and rescue approaches will be needed to determine whether ATF4 mRNA regulation under hypoxia, or under other stress conditions, specifically depends on p130.

In summary, our findings expand the current understanding of context-dependent ATF4 mRNA regulation by the p53 network. They indicate that p53 activation is associated with reduced ATF4 mRNA expression under selected stress conditions and that p21 and p130 contribute to this response in a stress-dependent manner. Our data further identify p130 as a candidate contributor to ATF4 mRNA regulation under hypoxia. ATF4-dependent stress adaptation has been implicated in tumor-cell survival under nutrient limitation, mitochondrial dysfunction, hypoxia, and exposure to anticancer agents. Therefore, the context-dependent regulation of ATF4 mRNA by p53, p21, and p130 described here may influence tumor sensitivity or resistance to p53-activating therapies and may help inform future combination strategies targeting p53 signaling together with ISR- or metabolism-dependent survival pathways. The molecular basis of these effects, including the contributions of transcription, mRNA stability, ATF4 protein regulation, and ISR activity, remains to be established.

## 5. Conclusions

In this study, we found that several commonly used chemotherapeutic agents that induce p53, as well as specific pharmacological p53 activation, reduce ATF4 mRNA expression. Experiments using the MDM2 inhibitor RG7388 and p53 knockdown indicate that p53 contributes to the reduction in ATF4 mRNA observed during mitochondrial dysfunction and glucose or glutamine deprivation, whereas ER stress overrides this response. Under these metabolic stress conditions, p21 and p130 also contributed to the decrease in ATF4 mRNA induced by RG7388 and doxorubicin.

In unstressed cells, p21, but not p130, contributed to RG7388-associated ATF4 mRNA suppression. In contrast, doxorubicin reduced ATF4 mRNA despite the depletion of p53, p21, or p130, indicating that additional pathways contribute to this response. Hypoxia also reduced ATF4 mRNA abundance, and this effect was partially relieved by p130 knockdown but not by the depletion of p53 or p21. These findings identify p130 as a candidate contributor to ATF4 mRNA regulation under hypoxic conditions.

Overall, our results reveal context-dependent associations between p53 activation, p21/p130 status, and ATF4 mRNA expression. They expand the current understanding of crosstalk between p53 signaling and the ATF4 branch of the integrated stress response. Further studies are required to define the underlying mechanisms and to determine how these changes in ATF4 mRNA affect ATF4 protein abundance, ISR activity, stress adaptation, and therapeutic responses in cancer cells.

## Figures and Tables

**Table 1:** Primers used for qPCR.

Primer	Sequence
ATF4-dir	CCAACAACAGCAAGGAGGATG
ATF4-rev	ATCCAACGTGGTCAGAAGGT
p21-dir	ACCATGTGGACCTGTCACTGT
p21-rev	TTAGGGCTTCCTCTTGGAGAA
TP53INP1-dir	CTGTGCATAACTCCTGCCCT
TP53INP1-rev	CACTTCTGTGCCCGTGAGTC
p130-dir	GCTACACGCTGGAGGGAAATGA
p130-rev	GTTTCCTTCCACTGTCCCTTTGC
TBP-dir	ACAGGAGCCAAGAGTGAAG
TBP-rev	AGGAGAACAATTCTGGGTTTG
TUBGCP2-dir	GACTGCGGAGAACATATTGTGA
TUBGCP2-rev	TCAATGTAGACCTCAGCCCC

## Data Availability

Raw data can be provided by the authors upon request.

## Supplementary Materials

The following supporting information can be downloaded at: https://www.mdpi.com/article/10.3390/cells15181713/s1, Figure S1: Upregulation of the mRNA expression of the p53 targets p21 and TP53INP1. mRNA levels of TP53INP1 (A–C), and p21 (D–F) in HCT116 cells with wild-type p53 (GFPsh); Figure S2: p53 activation by doxorubicin, topotecan, nutlin-3a, and RG7388 is retained in the presence of ATF4 activators; Figure S3: p53 knockdown completely prevents ATF4 downregulation by RG7388, but not by doxorubicin in the absence of ATF4 activators, in contrast to p21 and p130 knockdown; while knockdown of either p53, p21, or 130 prevents RG7388 and doxorubicin-induced ATF4 transcriptional suppression in the presence of the ETC inhibitors; Figure S4: (A–C) p53 activation by doxorubicin and RG7388 in cells with p53 (A), p21 (B), or p130 (C) knockdown. Protein levels of p53 and p21 in HCT116 cells with wild-type p53 (GFPsh) treated with doxorubicin (DXR), 250 nM RG7388 (RG) alone or in combination with 250 nM piericidin A (PRA), 2 µM myxothiazol + 1 mM uridine (MUR), were assessed using Western blotting. Cells were incubated with all compounds for 21 h. Vinculin served as a loading control. (D) Efficiency of p130 knockdown. mRNA levels of p130 in HCT116 cells expressing GFPsh or p130sh. Relative expression was normalized by GFPsh; Figure S5: p53 activation by doxorubicin and RG7388 has no effect on ATF4 mRNA levels under hypoxic conditions; knockdown of p53 or p21 does not affect ATF4 mRNA levels during hypoxia, whereas p130 knockdown alleviates p53-dependent ATF4 transcriptional suppression.

## Abbreviations

The following abbreviations are used in this manuscript:

5-FU	5-Fluorouracil
ATF4	Activating transcription factor 4
CDE	Cell cycle-dependent element
CDKN1A	Cyclin-dependent kinase inhibitor 1A
CHR	Cell cycle genes homology region
DHODH	Dihydroorotate dehydrogenase
DR5	Death receptor 5
DREAM	DP, RB-like, E2F4 and MuvB complex
E2F4	E2F transcription factor 4
eIF2α	Eukaryotic initiation factor 2 alpha subunit
ER	Endoplasmic reticulum
ETC	Electron transport chain
FBS	Fetal bovine serum
GADD34	Growth arrest and DNA damage-inducible protein 34
ISR	Integrated stress response
PALA	N-phosphonacetyl-L-aspartate
PTM(s)	Post-translational modification(s)
TBP	TATA-box binding protein
TP53INP1	Tumor protein p53-inducible nuclear protein 1
TTFA	Thenoyltrifluoroacetone
TUBGCP2	Tubulin gamma complex-associated protein 2
VEGF	Vascular endothelial growth factor
