# Supplementary material for: The p53-Associated Regulation of ATF4 mRNA Expression Involves p21 and p130 in a Stress-Dependent Manner
Source: Cells. 2026 Sep 20;15(18):1713. doi: 10.3390/cells15181713 (PMC13605123; doi:10.3390/cells15181713)
Supplement: Supplementary file 1 [file cells-15-01713-s001.zip › cells-4501937-supplementary.pdf]

Supplementary materials

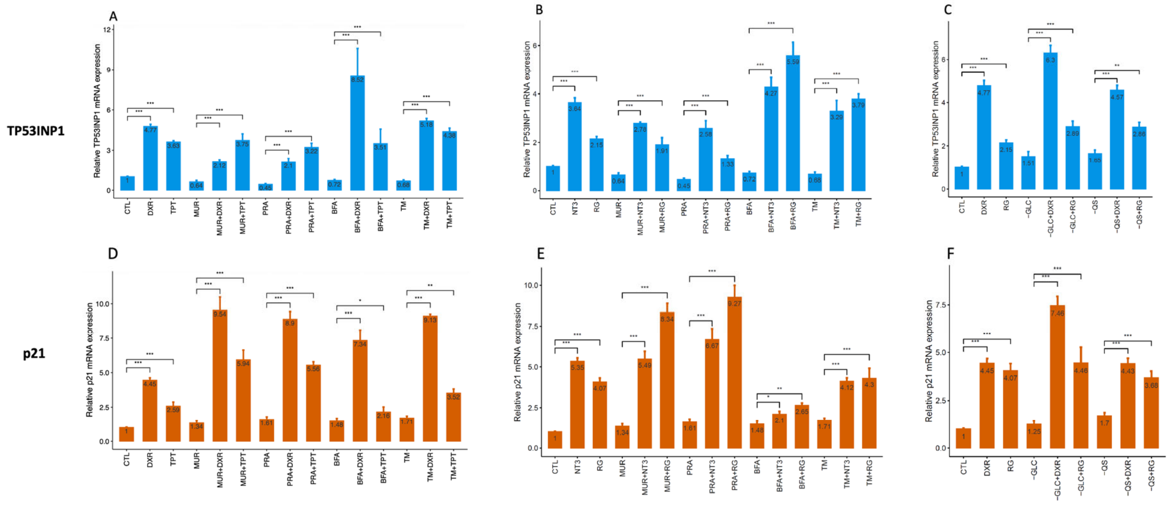

Supplementary Figure S1. Upregulation of the mRNA expression of the p53 targets p21 and TP53INP1. mRNA levels of TP53INP1 **(A-C)**, and p21 **(D-F)** in HCT116 cells with wild-type p53 (GFPsh). A-B,D-E. Cell were treated with 0.5 µM doxorubicin (DXR), 0.5 µM topotecan (TPT) **(A,D)**, 5 µM nutlin-3a (NT3), 250 nM RG7388 (RG) **(B,E)** alone or in combination with 250 nM piericidin A (PRA), 2 µM myxothiazol + 1 mM uridine (MUR), 5 µg/ml tunicamycin (TM), 25 µg/ml brefeldin A (BFA). C,F. Cell were treated with 0.5 µM doxorubicin (DXR) or 250 nM RG7388 (RG) either in the control medium (25 mM glucose, 4 mM glutamine), in the medium without glucose supplemented with 10% FBS, 4 mM glutamine, and 10 mM galactose (D), or in the medium without glutamine and FBS supplemented with 25 mM glucose. Cells were treated incubated with tunicamycin and brefeldin A for 5 hours and with all other compounds for 21 h. Relative expression was normalized by mock control (CTL). Results from three independent experiments with four biological replicates are presented as mean ± SEM. Two-way ANOVA, followed by the Tukey's post hoc test, was used. *$p < 0.05$, **$p < 0.01$, ***$p < 0.001$, NS – non-significant.

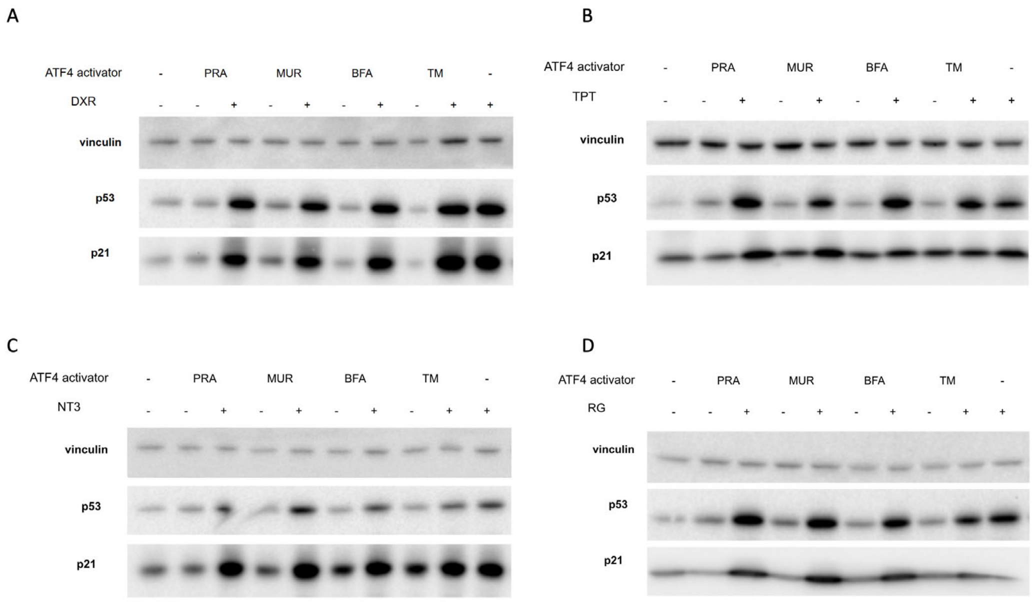

Supplementary Figure S2. p53 activation by doxorubicin, topotecan, nutlin-3a, and RG7388 is retained in the presence of ATF4 activators. Protein levels of p53 and p21 in HCT116 cells with wild-type p53 (GFPsh) treated with **(A)** 0.5 μM doxorubicin (DXR), **(B)** 0.5 μM topotecan (TPT), **(C)** 5 μM nutlin-3a (NTA), or **(D)** 250 nM RG7388 (RG) alone or in combination with 250 nM piericidin A (PRA), 2 μM myxothiazol + 1 mM uridine (MUR), 5 μg/ml tunicamycin (TM), and 25 μg/ml brefeldin A (BFA) were assessed using Western blotting. Cells were incubated with tunicamycin and brefeldin A for 5 hours and with the other compounds for 21 h. Vinculin served as a loading control.

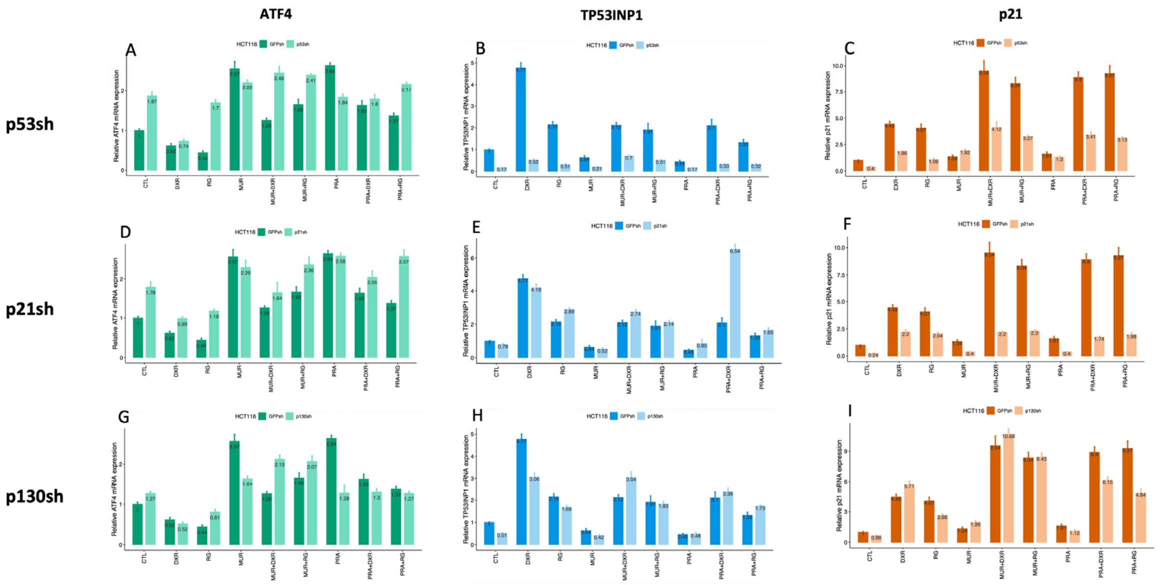

Supplementary Figure S3. p53 knockdown completely prevents ATF4 downregulation by RG7388, but not by doxorubicin in the absence of ATF4 activators, in contrast to p21 and p130 knockdown; while knockdown of either p53, p21, or 130 prevents RG7388 and doxorubicin-induced ATF4 transcriptional suppression in the presence of the ETC inhibitors. mRNA levels of ATF4 **(A,D,E)**, TP53INP1 **(B,E,H)**, and p21 **(C,F,I)** levels HCT116 cells expressing p53sh **(A-C)**, p21sh **(D-F)**, or p130sh **(G-I)** with 0.5 μM doxorubicin (DXR), 250 nM RG7388 (RG) alone or in combination with 250 nM piericidin A (PRA) or 2 μM myxothiazol + 1 mM uridine (MUR) for 21 h. Relative expression was normalized by GFPsh mock control (GFPsh CTL). Results from three independent experiments with four biological replicates are presented as mean ± SEM. Two-way ANOVA, followed by the Tukey's post hoc test, was used. *p < 0.05, **p < 0.01, ***p < 0.001, NS – non-significant.

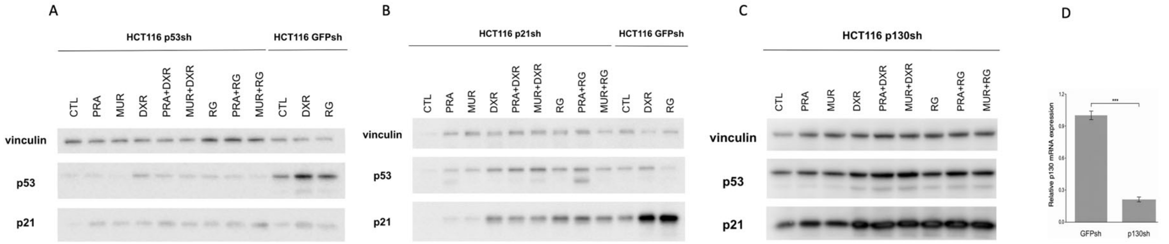

Supplementary Figure S4. A-C. p53 activation by doxorubicin and RG7388 in cells with p53 **(A)**, p21 **(B)**, or p130 **(C)** knockdown. Protein levels of p53 and p21 in HCT116 cells with wild-type p53 (GFPsh) treated with doxorubicin (DXR), 250 nM RG7388 (RG) alone or in combination with 250 nM piericidin A (PRA), 2 μM myxothiazol + 1 mM uridine (MUR), were assessed using Western blotting. Cells were incubated with all compounds for 21 h. Vinculin served as a loading control. D. Efficiency of p130 knockdown. mRNA levels of p130 in HCT116 cells expressing GFPsh or p130sh. Relative expression was normalized by GFPsh.

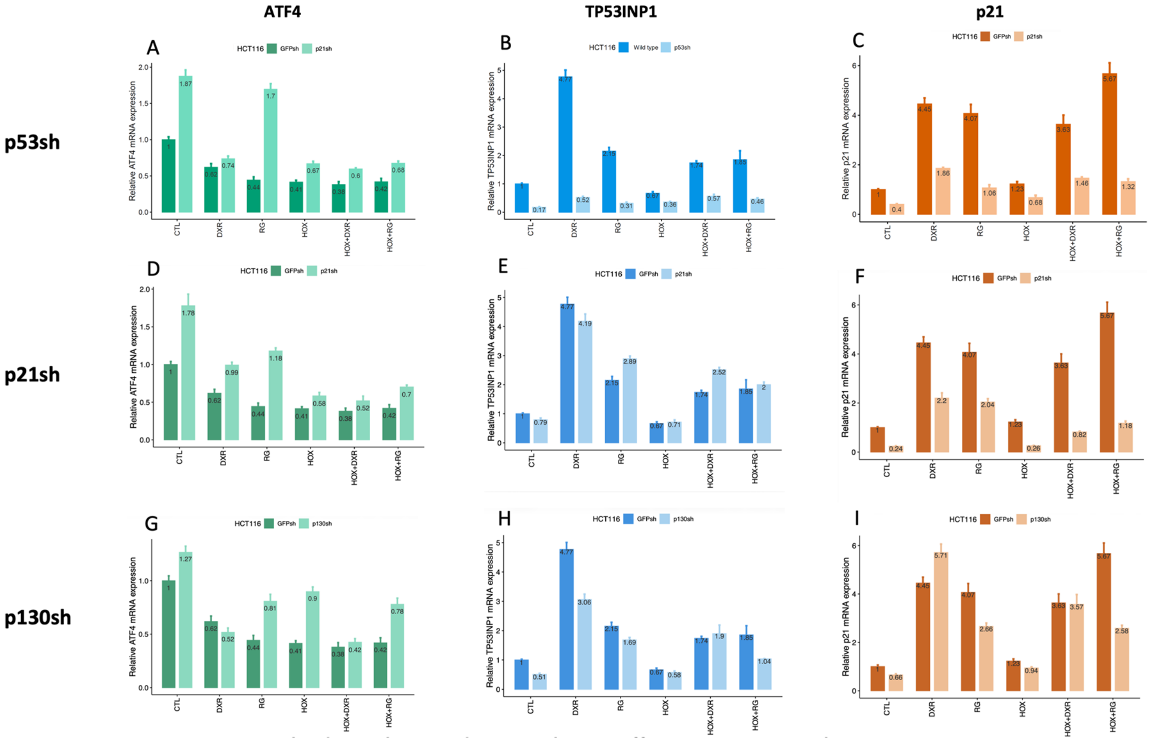

Supplementary Figure S5. p53 activation by doxorubicin and RG7388 has no effect on ATF4 mRNA levels under hypoxic conditions; knockdown of p53 or p21 does not affect ATF4 mRNA levels during hypoxia, whereas p130 knockdown alleviates p53-dependent ATF4 transcriptional suppression. mRNA levels of ATF4 **(A,D,G)**, TP53INP1 **(B,E,H)**, and p21 **(C,F,I)** levels in HCT116 cells expressing p53sh **(A-C)**, p21sh **(D-F)**, or p130sh **(G-I)** treated with 0.5 μM doxorubicin (DXR) or 250 nM RG7388 (RG) under normoxia or hypoxia (HOX) incubated for 21 h. Relative expression was normalized by GFPsh normoxic mock control (GFPsh CTL). Results from three independent experiments with four biological replicates are presented as mean ± SEM. Two-way ANOVA, followed by the Tukey's post hoc test, was used. *$p < 0.05$, **$p < 0.01$, ***$p < 0.001$, NS – non-significant.
